# Influence of PEG Stoichiometry on Structure-Tuned Formation of Self-Assembled Submicron Nickel Particles

**DOI:** 10.3390/ma11020222

**Published:** 2018-01-31

**Authors:** Bingxue Pu, Liping Wang, Heng Guo, Jian Yang, Haiyuan Chen, Yajun Zhou, Jin Yang, Bin Zhao, Xiaobin Niu

**Affiliations:** 1 State Key Laboratory of Electronic Thin Film and Integrated Devices, School of Materials and Energy, University of Electronic Science and Technology of China, Chengdu 610054, China; pubingxue@foxmail.com (B.P.); lipingwang@uestc.edu.cn (L.W.); guohenguestc@gmail.com (H.G.); yangjian.22@163.com (J.Y.); haiyuanchen@foxmail.com (H.C.); yjzhou9180@gmail.com (Y.Z.); jinwyj@outlook.com (J.Y.); 2 School of Architecture and Urban Planning, Shandong Jianzhu University, Jinan 250101, China

**Keywords:** Ni nanoparticles, solvothermal method, magnetic materials, microwave absorption

## Abstract

Self-assembled submicron nickel particles were successfully synthesized via the one-step surfactant-assisted solvothermal method. The impact of surfactant and reducing agent stoichiometry is investigated in this manuscript. Different morphologies and structures of Ni particles, including flower-like nanoflakes, hydrangea-like structures, chain structures, sphere-like structures, and hollow structures were prepared through different processing conditions with two parameters such as temperature and time. Based on scanning electron microscopy (SEM), X-ray diffraction (XRD), thermal gravimetric analysis (TGA) and vibrating sample magnetometry (VSM), the submicron nickel particles show good saturation magnetization and excellent thermal stabilities with a possible growth mechanism for the variety of the structure-tuned formation. Importantly, the microwave absorption properties of the submicron nickel particles were studied. The lowest reflection loss of Ni-P_9_/T_200_/H_15_ with a thin layer thickness of 1.7 mm can reach −42.6 dB at 17.3 GHz.

## 1. Introduction

Over the past few decades, the development of nanostructured materials has attracted substantial attention for many technological applications on account of their unique magnetic, optical, electrical, and catalytic properties [1,2,3]. Among them, metal nanomaterials are extremely attractive due to the possibility of controlling their multifunctionality and novel properties during synthesis [4]. Specifically, many research hotspots have been focused on the morphology-controlled synthesis of nickel (Ni) nanomaterials, since it is a necessary condition for achieving the objectives of nanoscience and nanotechnology [5]. As one of the nanostructured magnetic materials, Ni nanomaterials have been the focus of significant interests for researchers and display many characteristics such as chemical stability, large surface energy, and high surface area [6,7,8]. They are widely applied in a great many technological fields such as super capacitors, information storage, biotechnology and environmental protection [9,10,11]. Furthermore, Ni nanoparticles have attracted wide interests for their intriguing magnetic, catalytic, and microwave-absorbing properties under magnetic fields because of the abundance of Ni compared to other metals, which inspires researches on size and shape control of tunable nanostructures for many applications.

It is well known that the properties of nanoparticles are affected by their surface morphologies, crystal structures and dimensions. Different applications require different performances. Thus, it is important to study the properties of Ni nanoparticles using different process routes [12,13]. However, numerous successful chemical and physical methods have been used to fabricate the desired Ni architectures such as sonochemical synthesis, electrodeposition, electrochemical corrosion, and thermal decomposition [14,15,16,17]. These techniques usually involve the addition of various reducing agents, solvents, surfactants under complicated reaction conditions [18]. Therefore, many efforts have been directed toward using a facile and well-reproducible route to synthesize size- and shape-controlled Ni nanoparticles.

In previous work [19], we have reported a simple but effective one-step solvothermal method for the synthesis of pure nickel with different structures, such as flower-like Ni nanoflakes, hollow micrometer-sized Ni spheres/tubes. In this work, in order to further investigate the influence of poly (ethylene glycol) (PEG) stoichiometry on structure-tuned formation of Ni hierarchical architectures, we investigate the activity and selectivity of single PEG agent in the same one-step solvothermal method. At the same time, the structures and morphologies of the resultant Ni nanoparticles were characterized. We also find that their thermal and magnetic properties could be tuned by adjusting the main reaction parameters. The possible formation mechanism of Ni hierarchical architectures was also discussed. More importantly, we synthesized Ni metal nanoparticles with excellent microwave absorption properties in a simple, green and low-cost way, which performed superior microwave absorption with the lowest reflection loss of −42.6 dB at 17.3 GHz.

## 2. Experiment

### 2.1. Materials

Nickel chloride hexahydrate (NiCl_2_·6H_2_O, 99%), poly (ethylene glycol) (PEG, Mn  =  600, 99%), ethylene glycol (HOCH_2_CH_2_OH, EG, 99%), and anhydrous sodium acetate (CH_3_COONa, 99%) were supplied by Chengdu Kelong Chemicals Co. Ltd., Chengdu, China. All the chemicals were analytical purity and used without any further purification. 

### 2.2. Synthesis of Nickel Submicron Particles

A series of nickel nanoparticles were synthesized via a solvothermal method according to the previous work with slightly modification [19]. Typically, NiCl_2_·6H_2_O (2.97 g) and NaAc (9.0 g) were placed into 80 mL of EG solution in a three-neck flask, then adding a designed stoichiometry of PEG (e.g., 1.5 g). The mixtures were then stirred vigorously in an ultrasonic bath for 1 h at 60 °C that formed a light green solution. Then, the 80 mL above-mentioned solutions were sealed in a 100 mL Teflon-lined stainless-steel high-pressure autoclave, and maintained at 200 °C for 15 h. After natural cooling to room temperature, the products were washed several times with deionized water and alcohol, successively, and finally dried in a vacuum at 60 °C for 24 h. At last, the prepared Ni nanoparticles was termed as Ni-P_1.5_/T_200_/H_15_. Meanwhile, other Ni nanoparticles were prepared by adjusting the stoichiometry of PEG, solvothermal reaction temperatures and reaction times, which mark Ni-P_x_/T_y_/H_z_ (x means the weight of PEG, y is the reaction temperature and z represents reaction time). The detailed reaction parameters of the nickel nanoparticles are described in Table 1.

### 2.3. Characterization

The sizes and morphologies of the samples were observed by a JSM-6490LV scanning electron microscopy (SEM, JEOL, Tokyo, Japan). A Rigaku RINT2400 X-ray diffractometer used to analyze the phases of the samples (XRD, Rigaku, Tokyo, Japan). Thermal gravimetric analysis was performed in a TA Instruments Q50 (TGA, TA Instruments, New Castle, DE, USA). The magnetic properties of the nickel nanoparticles were measured by an IBHV-525 vibrating sample magnetometer (VSM, Riken Denshi, Tokyo, Japan). The microwave electromagnetic properties were investigated using a vector network analyzer (Agilent 8720ET, Agilent Technologies Inc., Santa Clara, CA, USA) in the frequency range of 0.5–18.0 GHz. 

## 3. Results and Discussion

### 3.1. Morphological Properties

**Figure 1:**
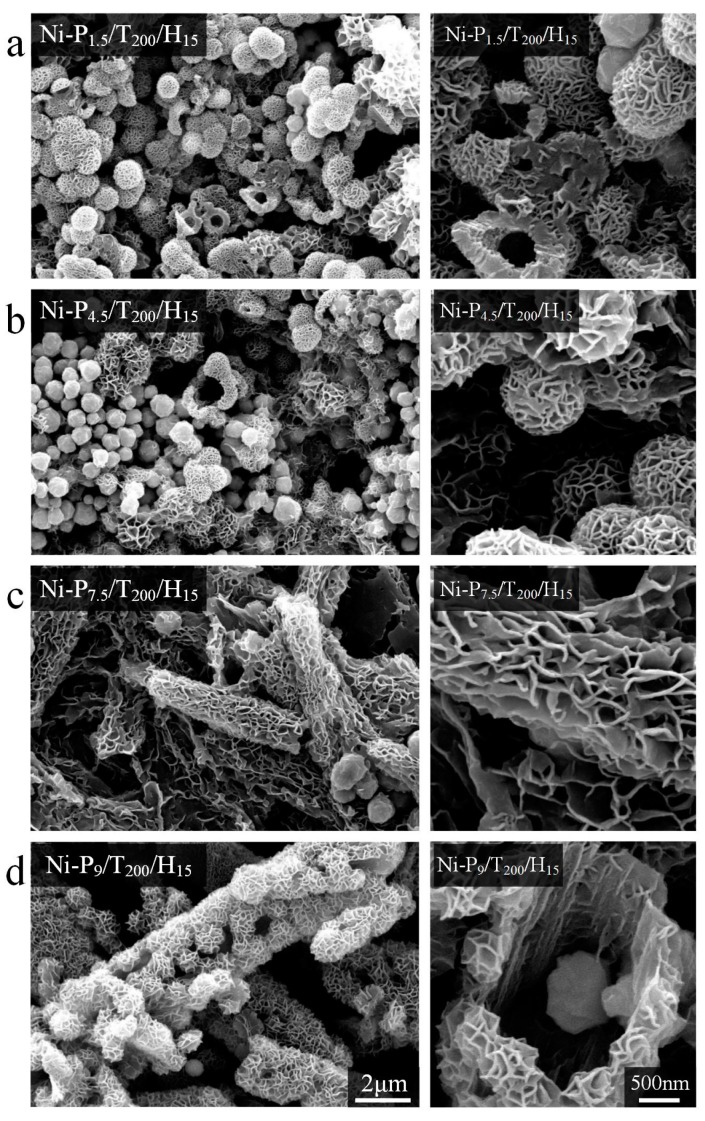
SEM images of Ni-P_1.5_/T_200_/H_15_ (**a**); Ni-P_4.5_/T_200_/H_15_ (**b**); Ni-P_7.5_/T_200_/H_15_ (**c**); and Ni-P_9_/T_200_/H_15_ (**d**) powders. The scale bars apply to all images in a column.

**Figure 2:**
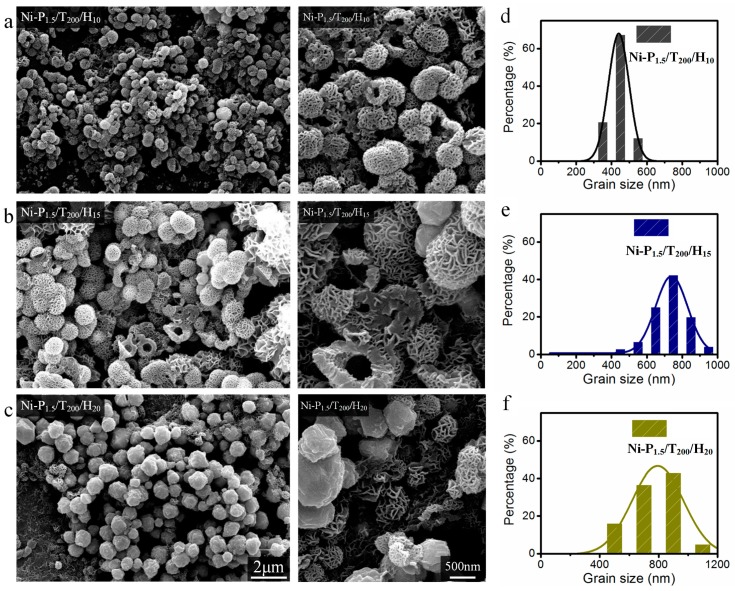
SEM images and corresponding particle size distributions of Ni-P_1.5_/T_200_/H_10_ (**a**,**d**), Ni-P_1.5_/T_200_/H_15_ (**b**,**e**), and Ni-P_1.5_/T_200_/H_20_ (**c**,**f**) powders. The scale bars apply to all images in a column.

In this solvothermal reaction system, PEG was used as reductant and template in the synthesis of Ni nanoparticles. Therefore, the influence of PEG stoichiometry on morphology of Ni nanoparticles was investigated by SEM. As can be seen from Figure 1a that sample (Ni-P_1.5_/T_200_/H_15_) is mainly composed of curly nanoflakes with hollow structures. With increasing of PEG, hydrangea-like submicron spheres (Ni-P_4.5_/T_200_/H_15_) are observed in Figure 1b. In Figure 1c, well-defined rod-like structures (Ni-P_7.5_/T_200_/H_15_) were formed. Magnified image shows that these rod-like structures are probably due to self-assembled with dense nanoflakes. In Figure 1d, there are massive hydrangea-like submicron hemispheres (Ni-P_9_/T_200_/H_15_) with a diameter of ca. 670 nm, formed tubular structures with a diameter ca. 3 μm. It can be observed that the structure of nanoparticles changes from hydrangea-like submicron spheres to tubular as the weight of PEG increases. It seems that the hydrophilic groups in PEG polymeric chain are adsorbed on the surface of nanoparticles to prevent the aggregation, and promote the 3D growth of Ni nanoparticles with reduced surface energy [20,21]. Moreover, the morphologies and structures of Ni nanoparticles are investigated by altering the reaction temperature and reaction time while keeping the other conditions constant. Figure 2 shows the morphologies of Ni-P_1.5_/T_200_/H_10_, Ni-P_1.5_/T_200_/H_15_ and Ni-P_1.5_/T_200_/H_20_ samples synthesized at 200 °C for 10, 15 and 20 h respectively. In addition, based on SEM images, the corresponding particle-size analysis obeys normal Gaussian distributions. In Figure 2a, we can see that the Ni-P_1.5_/T_200_/H_10_ spheres with hollow structures have some similarities with Ni-P_1.5_/T_200_/H_15_, but the average diameter of Ni-P_1.5_/T_200_/H_10_ (440 nm) is obviously smaller than that of Ni-P_1.5_/T_200_/H_15_ (730 nm). It can be deemed that with the increase of reaction time, the diameter of the nickel spheres becomes larger. While the reaction time extends to 20 h, almost all the nanoparticles (Ni-P_1.5_/T_200_/H_20_) have irregular sphere-like shape with an average diameter of ca. 770 nm, there are still a few flower-like nanospheres can be found in Figure 2c. With a closer inspection, the nanospheres have irregular radius. According to the mechanisms of aggregative growth [22], in the initial induction period, the size of the nanoparticles increase corresponds to the classical nucleation and growth rate. This regime may be followed with aggregative nucleation and growth. Small nanoparticles have higher mobility and collision frequency than large nanoparticles, owing to their large surface fractions and high energies. Therefore, the small nanoparticles are found to be more prone to merge than large nanoparticles [23]. We thus infer that as the reaction time increases the small nanoparticles tends to form large particles through mutual integration and thus have irregular radius.

**Figure 3:**
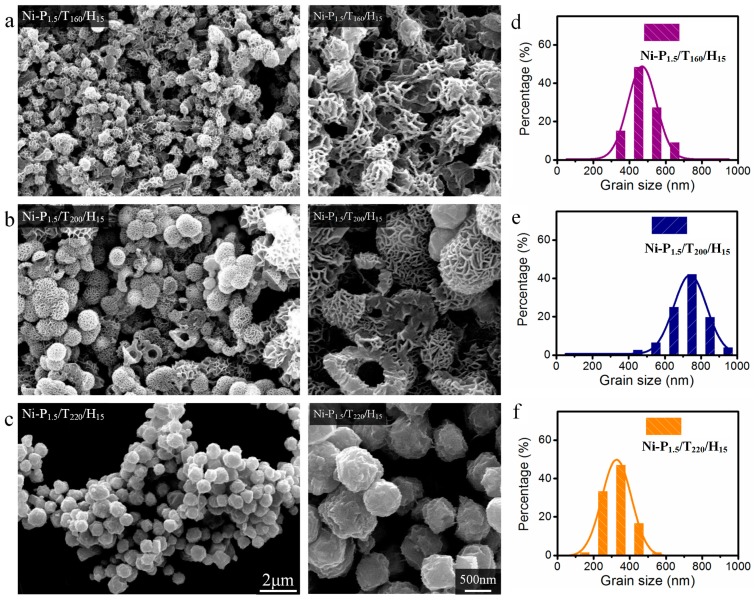
SEM images and corresponding particle size distributions of Ni-P_1.5_/T_160_/H_15_ (**a**,**d**), Ni-P_1.5_/T_200_/H_1__5_ (**b**,**e**) and Ni-P_1.5_/T_220_/H_15_ (**c**,**f**) powders. The scale bars apply to all images in a column.

The effect of reaction temperature on morphology was also discussed in this work. Figure 3a shows the representative SEM images of the Ni-P_1.5_/T_160_/H_15_ fabricated at 160 °C for 15 h. It is observed that most Ni-P_1.5_/T_160_/H_15_ nanoparticles are similar in shape with Ni-P_1.5_/T_200_/H_15_ with an average diameter of 470 nm. Meanwhile, it is seen that the Ni-P_1.5_/T_160_/H_15_ nanoparticles consist of interconnected and conglutinated petals. However, as the reaction temperature increases to 220 °C, the Ni-P_1.5_/T_220_/H_15_ nanoparticles aggregate together and possess the sphere-like structures. It is found that the Ni-P_1.5_/T_220_/H_15_ nanoparticles are close to regular submicron spheres with rough surface and with an average diameter of 660 nm (Figure 3c). Compared with Ni-P_1.5_/T_200_/H_15_, the increase of reaction temperature obviously leads to the reduction of the crystal size of Ni nanoparticles. As a result, the reaction temperature and time play a role in the particle size and morphology of Ni nanoparticles.

**Figure 4:**
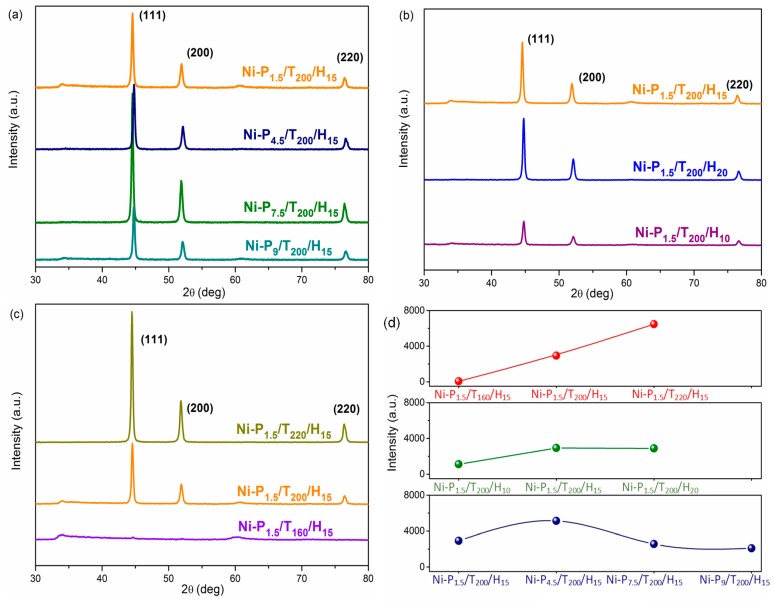
(**a**) XRD patterns of Ni-P_1.5_/T_200_/H_15_, Ni-P_4.5_/T_200_/H_15_, Ni-P_7.5_/T_200_/H_15_, and Ni-P_9_/T_200_/H_15_; (**b**) Ni-P_1.5_/T_200_/H_10_, Ni-P_1.5_/T_200_/H_15_, and Ni-P_1.5_/T_200_/H_20_; (**c**) Ni-P_1.5_/T_220_/H_15_, Ni-P_1.5_/T_200_/H_15_, and Ni-P_1.5_/T_160_/H_15_; (**d**) The intensity of XRD peaks of all samples.

In order to investigate the crystal structure crystal and phase of these samples, XRD patterns of the as-prepared Ni nanoparticles were conducted. As shown in Figure 4, most of the samples present characteristic diffraction peaks at 44.5°, 51.8° and 76.4°, corresponding to Ni (111), (200), and (220), which belong to the face centered cubic (FCC) structure in good consistent with the database of JCPDS Card (No. 04-0850) [24]. However, as for Ni-P_1.5_/T_160_/H_15_, the intensity of the characteristic diffractions peaks is weak, indicating that the lower temperature is adverse to reduce Ni^2+^ to Ni and form Ni crystals completely. According to the reported data (JCPDS card No. 04-0850), it can be identified that the Ni-P_1.5_/T_160_/H_15_ is nickel hydroxide [Ni(OH)_2_·0.75H_2_O]. This result indicates that Ni(OH)_2_·0.75H_2_O formed at above low reaction temperature is the precursor of Ni crystals, thus explains the reason that the morphology of Ni-P_1.5_/T_160_/H_15_ is similar to that of Ni-P_1.5_/T_200_/H_15_ [25,26,27]. The XRD patterns for Ni-P_1.5_/T_160_/H_15,_ Ni-P_1.5_/T_200_/H_10_ and Ni-P_1.5_/T_200_/H_15_ are further exhibited in Figure S1 and S2 (Supplementary Materials). As shown in Figure 4d, it is revealed that the intensity of diffraction peaks significantly increases as the reaction temperature increases, which indicates that the samples have higher crystallinity and better purity [19]. In addition, as the reaction time increases, the intensity of diffractions peaks increases. Interestingly, the peaks are observably enhanced with increasing of the PEG weight first, and then the intensity of the peaks is found to be decreasing with the weight of PEG increases. The variation in intensity of the peaks reveals that the PEG surfactant plays an important role in the fabrication of Ni particles, which is consistent with the SEM results.

### 3.2. Thermal Properties

**Figure 5:**
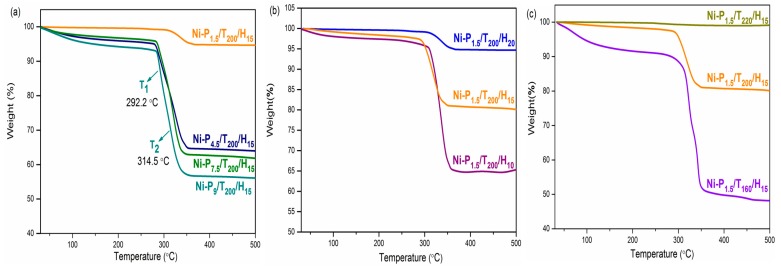
(**a**) TGA curves of Ni-P_1.5_/T_200_/H_15_, Ni-P_4.5_/T_200_/H_15_, Ni-P_7.5_/T_200_/H_15_, and Ni-P_9_/T_200_/H_15_; (**b**) Ni-P_1.5_/T_200_/H_10_, Ni-P_1.5_/T_200_/H_15_, and Ni-P_1.5_/T_200_/H_20_; (**c**) Ni-P_1.5_/T_220_/H_15_, Ni-P_1.5_/T_200_/H_15_, and Ni-P_1.5_/T_160_/H_15_.

The TGA spectra of various samples are performed and the results are shown in Figure 5 and Table 2. Almost all samples indicate that weight losses have two phases decomposition process with the maximum-rate decomposition temperatures of the first (T1) and second (T2) phases. Figure 5a shows the TGA curves of the Ni-P_1.5_/T_200_/H_15_, Ni-P_4.5_/T_200_/H_15_, Ni-P_7.5_/T_200_/H_15_ and Ni-P_9_/T_200_/H_15_ samples. At the first phase (below 300 °C) there is a slow decomposition rate due to the thermal decomposition of organic components in the samples [28]. The maximum decomposition temperatures of the second phase were at 319.2, 326.5, 320.1 and 314.5 °C respectively. In addition, the total weight losses at 500 °C were 19.9%, 36.1%, 38.2% and 43.9% respectively for the first group of samples. The second phase of decomposition process is due to the decomposition reaction of nickel. This decomposition process may be related to the crystallinity and structure of nickel [29]. These results indicate that the thermal stability of the samples decreases with the increasing addition of PEG. Additionally, the samples exhibit good thermal stabilities owing to the increasing of the reaction time and temperature, shown in Figure 5b,c. As is known to all, the magnetic properties of the nanomaterials are widely claimed to be highly dependent on the crystallinity, morphology, size and composition [30]. Therefore, these data reveal that thermal stability of the nickel particles was determined corresponding to the structures and appeared to be related to the PEG content.

### 3.3. Magnetic Properties

**Figure 6:**
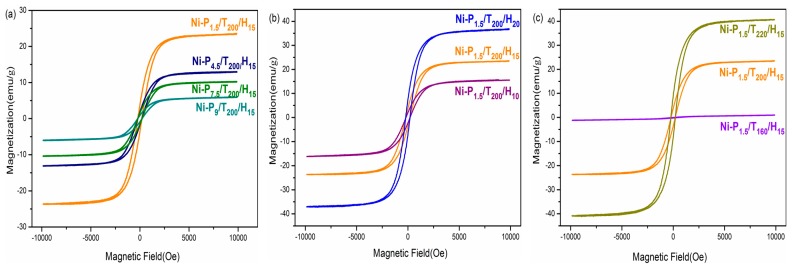
(**a**) Magnetization curves of Ni-P_1.5_/T_200_/H_15_, Ni-P_4.5_/T_200_/H_15_, Ni-P_7.5_/T_200_/H_15_, and Ni-P_9_/T_200_/H_15_; (**b**) Ni-P_1.5_/T_200_/H_10_, Ni-P_1.5_/T_200_/H_15_, and Ni-P_1.5_/T_200_/H_20_; (**c**) Ni-P_1.5_/T_220_/H_15_, Ni-P_1.5_/T_200_/H_15_, and Ni-P_1.5_/T_160_/H_15_.

The magnetic properties of the different samples (the magnetic value were referred to the content of only the metallic part) are measured in an applied magnetic field sweeping from −10,000 to 10,000 Oe, and shown in Table 2. The hysteresis loops in Figure 6a shows that the saturation magnetization (Ms) and coercive force (Hc) of Ni-P_1.5_/T_200_/H_15_, Ni-P_4.5_/T_200_/H_15_, Ni-P_7.5_/T_200_/H_15_, and Ni-P_9_/T_200_/H_15_ samples with different PEG content are 23.5, 13.0, 10.0, 5.9 eum·g^−1^ and 205.7, 200.8, 218.0, 222.2 Oe respectively. It is found that the Hc value was significantly improved relative to the bulk nickel (100 Oe), possibly due to its nanosized structure [31]. Interestingly, the Ms of Ni particles decreases along with the increasing content of PEG. It can be inferred that the surfactant affects the particle size, crystallinity and morphology of the nanoparticles, and thus affects the magnetic properties of the nanoparticles, which has a special structure and high order of shape anisotropy [32]. As shown in Figure 6b, the Ms values of Ni-P_1.5_/T_160_/H_15_, Ni-P_1.5_/T_200_/H_15_ and Ni-P_1.5_/T_220_/H_15_ samples are 15.9, 23.5 and 36.8 eum g^−1^, respectively, and the Hc values are 197.8, 205.7 and 206.8 Oe respectively. Clearly, the saturation magnetization and coercivity increase with the increase of reaction time. In similarity, it can be seen from Figure 6c that with the increase of the reaction temperature, the saturation magnetization and coercivity are also enhanced. It can be inferred that the reaction of temperature and time have a big impact on the crystallinity of the nanoparticles, which is corresponding to results of the SEM, XRD and TGA investigation.

### 3.4. Microwave Absorption Properties

**Figure 7:**
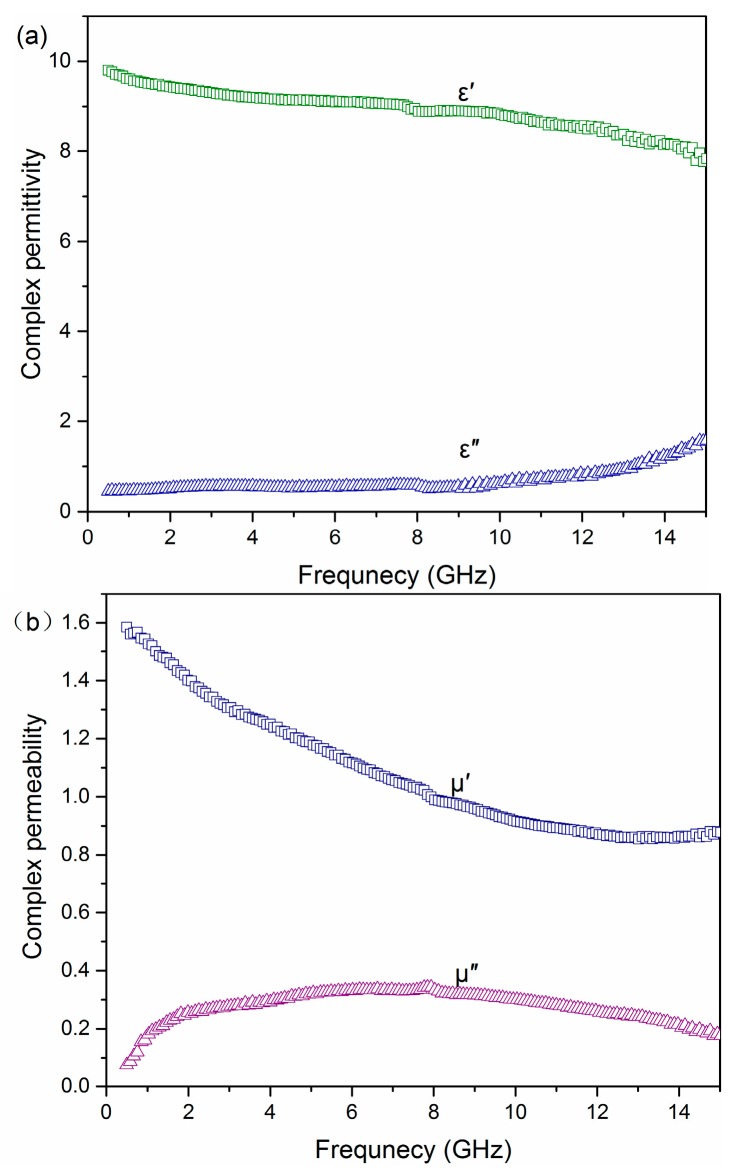
Electromagnetic parameters of 25 wt % paraffin Ni-P_9_/T_200_/H_15_ sample. (**a**) Complex permittivity and (**b**) Complex permeability.

It is generally known that the microwave absorption properties of materials are reflected by the value of the reflection loss (RL), which is closely related to the complex permittivity (ε′, ε′′), permeability (μ′, μ′′) as well as the thickness of absorber layer. The complex permittivity (ε′, ε′′) and permeability (μ′, μ′′) of the Ni-P_9_/T_200_/H_15_ with 25 wt % paraffin are shown in Figure 7a, respectively. With the increase of frequency, the ε′ value of Ni-P_9_/T_200_/H_15_ gradually decreases from 9.8 to 7.0, then stabilized. Meanwhile, ε′′ increases slowly from 0.5 to 1.6. When the frequency is relatively low, interfacial polarization can be induced, but with the increase of frequency, it cannot keep up with the pace of the alternating electric field [33]. Thus, as the frequency increases, relaxation polarization loss and electric conductance loss increase, which determines the increase of dielectric loss [34]. This explains why ε′′ increases while ε′ decreases as the frequency increases. In Figure 7b, it is clearly that the real permeability µ′ first decreases in the frequency range of 0.5–14 GHz, and then followed by a slight increase. The decline of µ′ is ascribed to the hysteresis of domain-wall motion and rotation as the frequency increases [35]. In contrast, the imaginary permeability µ′′ displays a wide peak in the frequency range of 2.5–15 GHz due to the domain-wall resonance and relaxation loss [36].

**Figure 8:**
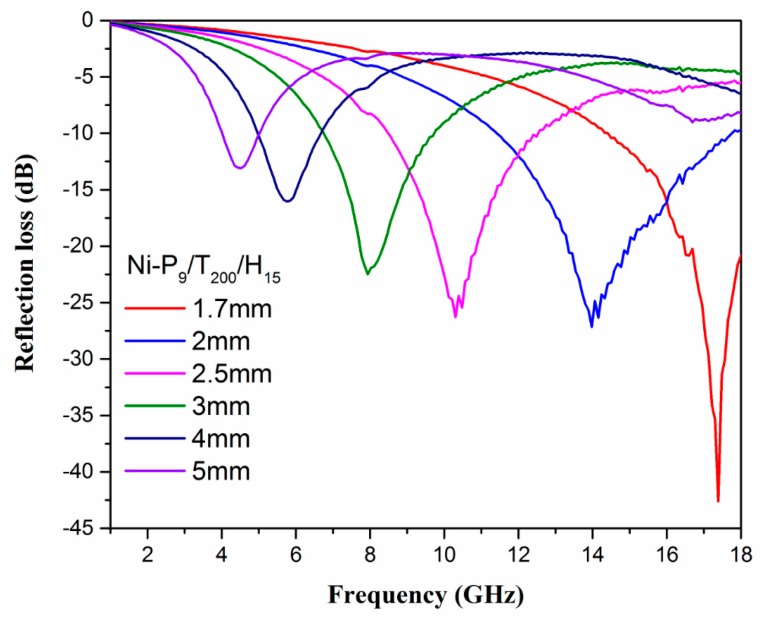
The calculated reflection losses of Ni-P_9_/T_200_/H_15_ with thicknesses of 1.7–5 mm.

**Figure 9:**
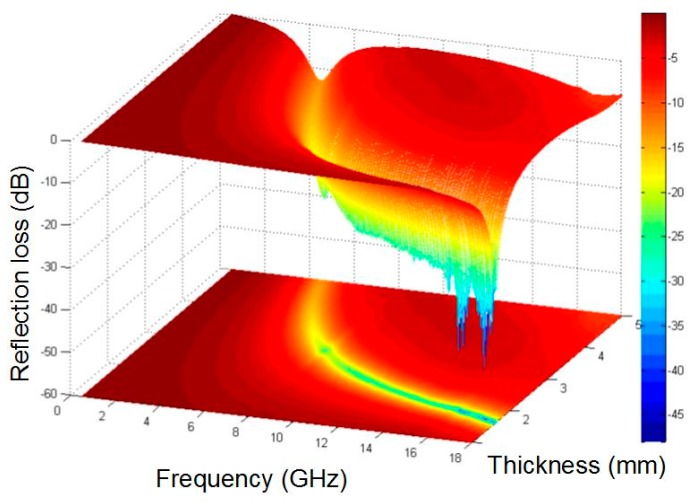
The calculated three-dimensional representations of reflection losses of Ni-P_9_/T_200_/H_15_ sample.

According to established model for single-layer plane-wave from the transmission line theory [37], the reflection loss (RL) can be calculated by the following equations:

(1) $$\mathrm{RL}\;(\mathrm{dB})=20\log\left|\frac{Z_{\mathrm{in}}-Z_{0}}{Z_{\mathrm{in}}+Z_{0}}\right|$$

(2) $$z_{\mathrm{in}}=z_{0}\sqrt{\frac{\mu_{r}}{\varepsilon_{r}}}\tan h\left(j\frac{2\pi \mathrm{fd}}{c}\sqrt{\mu_{r}\varepsilon_{r}}\right)$$

where Z_in_ is the normalized input characteristic impendence, Z_0_ is the free space impendence, f is the frequency of microwaves, d is the thickness of absorber layer, and c is the speed of light. ε_r_ and μ_r_ can be obtained by following equations ε_r_ = ε′ − jε′′ and μ_r_ = μ′ − jμ′′. The calculated results of RL of the Ni-P_9_/T_200_/H_15_ are shown in Figure 8. Moreover, the calculated reflection losses of pure paraffin is presented in Figure S3 (Supplementary Materials). When the thickness of absorber layer is 2, 2.5, 3, 4 and 5 mm, the corresponding minimum RL peak value is −27 dB at 13 GHz, −26 dB at 10.3 GHz, −22.5 dB at 7.9 GHz, −16 dB at 5.7 GHz and −9 dB at 16.8 GHz, respectively. It can be observed that the absorption frequency region shifts from low frequency to high frequency and its corresponding peak intensity increases as the absorber layer thickness increases. Particularly, the minimal RL of Ni-P_9_/T_200_/H_15_ is −42.6 dB at 17.3 GHz with 1.7 mm thickness of absorber layer, demonstrating superior electromagnetic microwave absorption properties in the Ku-band (12.0–18.0 GHz). In addition, Figure 9 shows the calculated three-dimensional representations of the RL for Ni-9 sample. It is clear that the value of the RL of Ni-P_9_/T_200_/H_15_ in the frequency range of 6–18 GHz is less than −20 dB, and the value of the reflection loss at 1.7 mm absorber layer is less than −40 dB. Although the RL peak value decreases as the absorber layer thickness increases, it has a wide absorption band. It is worth noting that if the value of the reflection loss is less than −20 dB, the equivalent of 99% of the electromagnetic wave will be absorbed [38,39]. More importantly, each absorber layer thickness of Ni-P_9_/T_200_/H_15_ corresponds to distinctly different absorption peak. This allows potential application in the notch filter [40], which is a sort of special electromagnetic shielding device that can rapidly attenuate the input signal at a certain frequency. The above results indicate that the Ni-P_9_/T_200_/H_15_ sample is an ideal microwave absorbing material with multi-band and strong absorption performance, which has extensive application.

## 4. Conclusions

In conclusion, self-assembled submicron Ni particles with different morphologies and structures were successfully prepared through a simple one-step surfactant-assisted solvothermal method. The morphologies of Ni particles were studied, indicating that the PEG as a surfactant plays an important part in the fabrication of Ni particles with the impact of stoichiometry. Meanwhile, different morphologies and structures of Ni particles including flower-like nanoflakes, chain structures, sphere-like structures, and hollow structures were obtained through altering reaction temperature and time. Importantly, these Ni particles exhibit excellent thermal stabilities with a possible growth mechanism for the variety of the structure-tuned formation. Additionally, the as-obtained Ni particles exhibit good saturation magnetization and high coercivity values as a result of the unique structures. Furthermore, the Ni-P_9_/T_200_/H_15_ sample exhibits excellent microwave electromagnetic properties. The absorbing layer with thickness of 1.7 mm possesses the minimum RL of −42.6 dB at 17.3 GHz. This one-pot synthesize method offers a practical, facile and economical way for preparations of Ni particles uniformly, and it is expected to have potential applications in commercial and industrial production of Ni particles.

## Figures and Tables

**Table 1:** The detailed reaction parameters of the nickel nanoparticles.

Samples	Mass of PEG (g)	Temperature (°C)	Time (h)	Crystal Size (nm)
Ni-P_1.5_/T_200_/H_15_	1.5	200	15	730
Ni-P_4.5_/T_200_/H_15_	4.5	200	15	640
Ni-P_7.5_/T_200_/H_15_	7.5	200	15	-
Ni-P_9_/T_200_/H_15_	9.0	200	15	670
Ni-P_1.5_/T_200_/H_10_	1.5	200	10	440
Ni-P_1.5_/T_200_/H_20_	1.5	200	20	770
Ni-P_1.5_/T_160_/H_15_	1.5	160	15	470
Ni-P_1.5_/T_220_/H_15_	1.5	220	15	660

**Table 2:** The thermal and magnetic properties of the as-prepared samples.

Samples	Ms (eum/g)	Mr (eum/g)	H_c_ (Oe)	T_1_ (°C)	T_2_ (°C)
Ni-P_1.5_/T_200_/H_15_	23.5	3.7	205.7	319.24	-
Ni-P_4.5_/T_200_/H_15_	13.0	1.8	200.8	291.4	326.5
Ni-P_7.5_/T_200_/H_15_	10.2	1.6	218.0	292.2	320.1
Ni-P_9_/T_200_/H_15_	5.9	0.87	222.2	292.2	314.5
Ni-P_1.5_/T_200_/H_10_	15.9	2.1	197.8	336.0	-
Ni-P_1.5_/T_200_/H_20_	36.8	7.4	206.8	337.6	-
Ni-P_1.5_/T_160_/H_15_	1.1	0.08	185.6	252.5	280.3
Ni-P_1.5_/T_220_/H_15_	40.7	8.5	216.6	322.5	341.6

## Supplementary Materials

The following are available online at http://www.mdpi.com/1996-1944/11/2/222/s1, Figure S1: XRD patterns of Ni-P1.5/T160/H15, Figure S2: XRD patterns of Ni-P1.5/T200/H10 (a) and Ni-P1.5/T200/H15 (b). Figure S3: The calculated reflection losses of pure paraffin with thicknesses of 5 mm.
