# Supplementary material for: Influence of PEG Stoichiometry on Structure-Tuned Formation of Self-Assembled Submicron Nickel Particles
Source: Materials (Basel). 2018 Jan 31;11(2):222. doi: 10.3390/ma11020222 (PMC5848919; doi:10.3390/ma11020222)
Supplement: Supplementary file 1 [file materials-11-00222-s001.pdf]

Supporting Information

Influence of PEG stoichiometry on structure-tuned formation of self-assembled submicron nickel particles

Bingxue Pu[1], Liping Wang[1], Heng Guo[1], Jian Yang[1], Haiyuan Chen[1], Yajun Zhou[1], Jin Yang[1], Bin Zhao[2*] and Xiaobin Niu[1*]

[1] School of Micro-electronics and Solid-state Electronics, State Key Laboratory of Electronic Thin Film and Integrated Devices, University of Electronic Science and Technology of China, Chengdu 610054, China

[2] School of Architecture and Urban Planning, Shandong Jianzhu University, Jinan, 250101, China

E-mail: xbniu@uestc.edu.cn, zbkite@sdjzu.edu.cn.

Fig.S1 shows the XRD patterns of Ni-$P_{1.5}/T_{160}/H_{15}$, the samples have characteristic diffraction peaks at 11.3 °,22.7 °, 34.4 °and 45.9 °corresponding to Ni (003), (006),(012) and (110), which match well with the values of the FCC phase α*-$Ni(OH)_2 \cdot 0.75H_2O$ (JCPDS Card No. 04-0850).

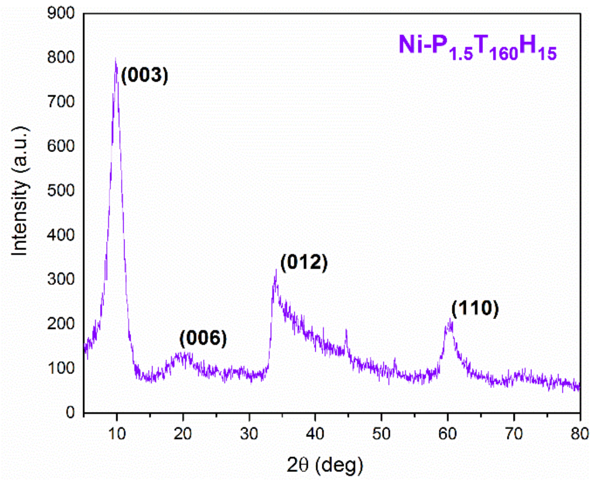

Fig.S1 XRD patterns of Ni-$P_{1.5}/T_{160}/H_{15}$.

Fig.S2 shows the XRD patterns of Ni-$P_{1.5}/T_{200}/H_{15}$ (a), and Ni-$P_{1.5}/T_{200}/H_{15}$(b). The XRD patterns indicate that the Ni-$P_{1.5}/T_{200}/H_{10}$ and Ni-$P_{1.5}/T_{200}/H_{15}$ contain a small amount of nickel hydroxide.

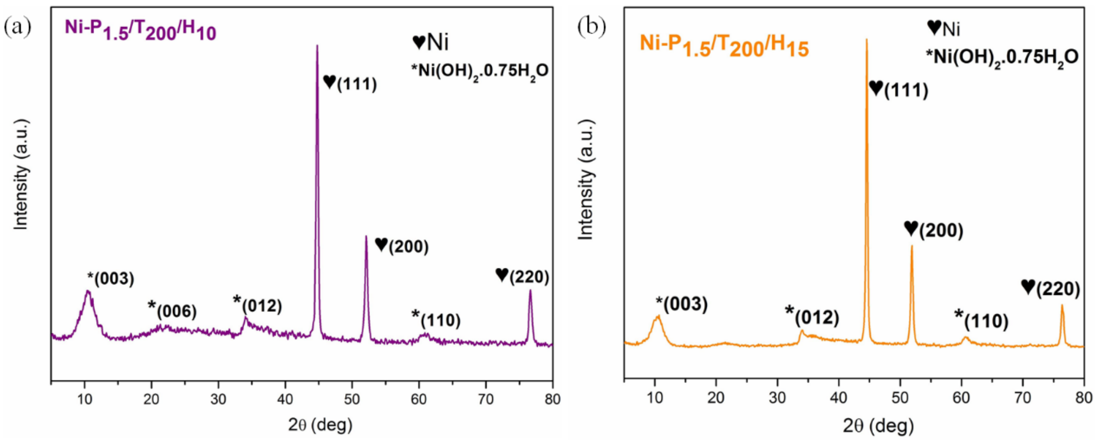

Fig.S2 XRD patterns of Ni-$P_{1.5}$/T_{200}/H_{10} (a) and Ni-$P_{1.5}$/T_{200}/H_{15} (b).

Fig.S3 shows the calculated reflection losses of pure paraffin with thicknesses of 5 mm. It can be inferred that pure paraffin can hardly cause any effect on the electromagnetic properties of the Ni-P_9/T_{200}/H_{15}.

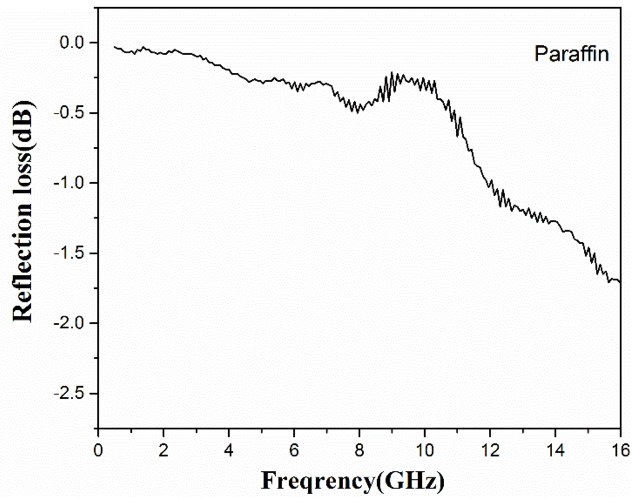

Fig.S3 The calculated reflection losses of pure paraffin with thicknesses of 5 mm.
